# Re: Dipyridamole reduces penile apoptosis in a rat model of post-prostatectomy erectile dysfunction

**DOI:** 10.1590/S1677-5538.IBJU.2018.0016

**Published:** 2018

**Authors:** Shanzun Wei, Ming Ma, Changjing Wu, Botao Yu, Jiuhong Yuan

**Affiliations:** 1 Andrology Laboratory, Westchina Hospital, SiChuan University, China; 2 Department of Urology, Westchina Hospital, SiChuan University, China

*To the editor,*

We read with interest the recent in vivo research in attempt to recover erectile dysfunction after radical prostatectomy accomplished by Omer kutlu et al. (1). They apply dipyridamole treatment in animal model of cavernous nerve crush injury. While they successfully unveiled that dipyridamole reduces apoptosis indices and TGF-β1 level in corpus cavernous tissue, they failed to establish improvement in ICP value-the golden standard of erectile function.

The author reckons the short period of treatment may result in the negative ICP difference between vehicle and treatment group. As to the modelling, 15 days in current study is sufficient for nerve crush injury to induce erectile dysfunction. Mullerad et al. previous revealed that decline in ICP may be detected 3 days after BCNI surgery, but no difference was observed in 10 days and 28 days post-surgery (2). However, Chan-Ho lee et al. compared the prognoses of post BCNI treatment between 4 weeks and 8 weeks after BCNI surgery. They discovered that 8 weeks of post BCNI treatment is more beneficial in ameliorating ICP declination and reducing cavernous SMC apoptosis than the 4 weeks regimen (3).

Furthermore, another ED etiological factor followed by PR surgery is operation related neurapraxia, which may result from mechanical traction, ischemia and focal inflammation (4). The adjacency predisposes the vulnerability of cavernous nerve in the RP surgery. Whereas, the most exquisitely performed nerve sparing-PR surgery may still render the possible occurrence of neurapraxia with impaired ED episode and is up to 18-24 months (5). In the current research, 15 days of treatment in rats merely equals to a rehabilitation program of 1 year for humans. This may partially explain limited ICP restoration achieved by the author in the15 days treatment (6). And we believe that if the author has extended the treatment period, a more pronounced therapeutic effect may thus be shown.

Transperitoneal anaesthesia with ketamine and xylazine mixture is valid and consistent in each group. However, we have previously assessed the impact of anesthesia on ICP values of normal rats and discovered that ICP value in rats anesthetized with inhalation was higher than rats anesthetized transperitoneally (7). Inhalation anaesthesia is more prompt in controlling anaesthetic depth control and regulating vital signs. It did not manifest with significant different vital signs and oxygen saturation compared to transperitoneal anaesthesia in rats. Also, it is suggested that inhalation anaesthesia may lead to steadier physiological state, provide sustainable and adequate anaesthesia depth that is advantageous in achieving valid and consistent ICP value. It can also reduce the risk of anaesthesia induced casualty. Li also believes that local anaesthetic effect could not be ruled out in transperitoneally anesthetized, and this may be another factor for the lower ICP.

In conclusion, we believe if the authors extended their treatment period and switched the anaesthesia, not only they would have achieved better apoptosis index, but also would have shown improvement in erectile functional index. For their discovery resembles to Karaguzel's research (8). Karaguezel applied dipyridamole in reducing acute penile ischemic and reperfusion injury in priapism. Though they did not focus on the dipyridamole's effect on reduce cavernous tissue apoptosis nor restoration post injury induced erectile function impairment. Both studies unveiled a promising therapeutic role of dipyridamole in reducing acute and chronic ischemic impact and disclosed a promising future of dipyridamole in urology clinic application.
